# Investigating the drying characteristics of *Anthocephalus chinensis* (Lam.) A. Rich ex Walp wood

**DOI:** 10.1016/j.heliyon.2022.e10360

**Published:** 2022-08-19

**Authors:** Devashish Kumar Ghosh, Md. Azharul Islam, Rupak Kumar Ghosh, Santosh Mazumdar, Atanu Kumar Das

**Affiliations:** a Forestry and Wood Technology Discipline, Khulna University, Khulna 9208, Bangladesh; b Bangladesh Forest Research Institute, Chittagong 4211, Bangladesh; c University of Chittagong, Chittagong 4331, Bangladesh; d Department of Forest Biomaterials and Technology, Swedish University of Agricultural Sciences, SE-90183 Umeå, Sweden

**Keywords:** Kiln drying, *A. chinensis*, Drying schedule, Drying defect, Drying quality

## Abstract

*Anthocephalus chinensis* (Lam.) A. Rich ex Walp is widely used as raw materials in particleboard and match industries in Bangladesh. The current study aimed to identify the drying characteristics of *A. Chinensis* wood for succeeding industrial usages. A compartment kiln dryer (heat and vent dryer) was used in this study. The drying characteristics and drying quality of *A. Chinensis* wood were measured. The boards reached 6–10% moisture content in 13 days from their green condition. The total proportions of the check, twist, and collapse in boards were 22.5, 32.5, and 7.3%, respectively. The volumetric shrinkage was 21.67%. Based on this study, further study may help to develop a complete drying schedule of *A. Chinensis* wood with fewer drying defects for application at industrial level.

## 1 Introduction

The utilization of wood as an engineering material depends on the shape stability [1]. It can help to use wood as an alternative to concrete and steel [2]. In addition, strength properties are increased when the wood is dried from green to below the fiber saturation point [1, 3]. Drying also improves adhesive compatibility and increases resistance to biodegradation of wood [1].

Wood contains a huge amount of moisture in its green condition [4, 5]. The moisture content depends on the wood species and the season of the harvesting [4]. The removal of moisture is the prerequisite for the processing of wood [5]. Due to its ordinary physical and mechanical properties, proper drying before use is the main factor in its efficient and rational utilization [6]. The modern wood industry uses mainly air-drying, kiln drying, and vacuum drying to reduce the wood moisture content [7, 8]. Alternative methods, i.e., supercritical drying, higher temperature, low pressure, microwave, etc. have been developed to reduce the drying time [9, 10]. The main objectives of wood drying programs include minimizing energy consumption, expanding drying rate, significant quality, and finally lessening drying costs [11].

The drying ability of wood depends on the initial moisture content and density of wood and drying condition [11]. The drying of wood also causes various defects due to transferring heat and mass during drying [6]. The differences in temperature and humidity during the drying of wood affect the grade of the final products. Various drying schedules result in diverse quantities of deformations, stresses, and crack development [12]. The controlled drying and use of pre-treatment methods help to reduce the drying defects of wood. A proper way of drying can diminish warping, shrinkage, and checking of wood [2, 5]. However, drying duration and controlled parameters depend on species.

Wood demand is increasing gradually in Bangladesh [13, 14, 15, 16, 17, 18] and people are being interested in fast-growing species to meet the demand for wood. *Anthocephalus chinensis* (Lam.) A. Rich ex Walp belongs to the family of Rubiaceae is commonly found all over the Indian subcontinent including Bangladesh, the Indo-Malayan region, and Borneo to the Philippines and New Guinea [19]. As a fast-growing species, it grows rapidly in the first 6–8 years with a broad crown and straight cylindrical bole attaining a height of 45 m having 100–160 cm in trunk [20]. It is used in the match factory and particleboard industry [20]. The improvement of wood properties may help its application in furniture industry and construction purposes. The proper drying schedule of *A. chinensis* wood is very necessary for its commercial usages.

Therefore, the present study aimed to understand a drying scheme of *A. chinensis* wood and to investigate the drying effect on wood quality for further development of drying schedule intending future commercial usages. Based on the findings, the possible recommendations for improving the drying quality had been put for further study in detail.

## 2 Materials and methods

### 2.1 Preparation of raw material

Around 18-year-old straight and cylindrical *A. chinensis* trees with 24 m in bole height and 0.3 m in diameter (DBH) were sourced from Savar (23° 51′ 30.0024″ N and 90° 16′ 0.0120″ E), Dhaka, Bangladesh. A 3 m log was selected from the base of the tree for converting into boards. From the logs, boards in a dimension of 2183 × 270 × 35 mm were prepared and a total of 220 boards were used in this study. The boards were prepared after immediate felling of the trees and put them into the dryer for drying. The presence of sapwood and heartwood was not considered in the present study because the understanding of general drying behavior was the main objective. The basic density of wood was determined based on dry weight and saturated volume following the standard of SCAN-CM 43:95 [21].

### 2.2 Drying process

A compartment kiln drier was used to investigate the drying characteristics of the sample in this study. Drying schedule followed was according to the schedule used in the industry where this species is processed and was done to understand the drying characteristics of this species. One drying schedule was used identifying the drying behaviour in this study, and it was dried based on the target moisture content. The low temperature was used after eight days to reduce the drying defects. Size of the kiln room was 9.1 × 4.9 × 6.1 m. There were five loads in total and each load had 44 samples of *A. chinensis* lumber. The lumbers were stacked horizontally with the sticker spacing of 30 cm and the sticker was 5 cm in width and 2.5 cm in thickness. The entire stack was seasoned using a program setting until the total stack was decreased to required moisture content (MC). Temperature and relative humidity (RH) were measured every 24 h. Electrical fans were operated for rapid motion of ventilation. Variable speed fans were used to maintain the airflow. The continuous airflow was used with an average of 2.5 m/s along the completely drying period. The measured values of wet and dry bulb temperature, relative humidity, and target moisture content setting during drying are shown in Table 1.

**Table 1:** Drying schedule for *A. chinensis*.

Drying Schedule					
Time (Day)	Relative Humidity (%)	Dry bulb Temp (°C)	Wet bulb Temp (°C)	Moisture content (%)	Remarks
0	-	-	-	120 (±5)	Initial moisture content
1	80 (± 2)	40 (± 1)	36 (± 2)	60 to50	After 1 day
2	80 (± 5)	45 (± 2)	40 (± 2)	50 to47	-
3	76 (± 3)	46 (± 2)	42 (± 1)	47 to45	-
4	61 (± 1)	50 (± 3)	42 (± 2)	45 to40	
5	65 (± 2)	55 (± 1)	42 (± 3)	40 to34	-
6	56 (± 3)	55 (± 3)	42 (± 1)	34 to30	
7	52 (± 4)	60 (± 2)	42 (± 2)	30 to27	-
8	56 (± 2)	60 (± 1)	42 (± 3)	27 to20	-
9	65 (± 3)	55 (± 2)	42 (± 2)	20 to18	-
10	61 (± 1)	55 (± 3)	42 (± 1)	18 to15	-
11	56 (± 2)	45 (± 1)	42 (± 3)	15 to12	-
12	40 (± 5)	45 (± 3)	42 (± 1)	12 to10	-
13	30 (± 3)	40 (± 2)	36 (± 2)	10 to 6	Final moisture

Note: Values in parentheses indicate the standard deviation and any stabilization stage was not applied.

### 2.3 Drying characteristics

Drying characteristic was measured based on the MC reduction. At first, representative samples were collected during preparing the boards to determine the initial moisture content for each load. Then, the MC was measured every 24 h until reaching a target moisture content of 6–10%. For each time, five boards were used to measure the MC from each load. To measure the moisture gradient of the final dried boards, MC of the samples from three positions, i.e., two ends and middle of was determined and the samples were taken from five boards randomly of each load. The MC was determined using the weighing method and it was calculated based on oven dry weight basis.

### 2.4 Drying quality

Drying quality was analysed based on the drying defects and dimensional stability. Twist, checks, and collapse were measured to investigate the drying defects. The check was determined with a Mitutoyo digital gauge following the methods described by Korkut & Guller [11]. The little check considered for ≤1.0 mm in depth occurred in the dried sample while ≥1.0 mm in depth was in the group of severe checks for this study. Displacement in deformation gauges (LVDTs) technique was used to analyse the twist based on the procedure explained elsewhere [2]. The distortion of >0^o^ was considered as a twist for this study. The collapse was checked using the method defined elsewhere [22]. In this study, physical appearance, i.e., grooves or corrugations of the boards was considered to measure the collapse. Shrinkage was determined to identify the dimensional stability of the *A. chinensis* board. Width, thickness, and volumetric shrinkage were analysed in this study.

### 2.5 Statistical analysis

Statistical analysis was performed by the spreadsheet-2016. Standard deviation and standard error were measured for this study.

## 3 Results and discussions

### 3.1 Drying characteristic

#### 3.1.1 Drying time

**Figure 1:**
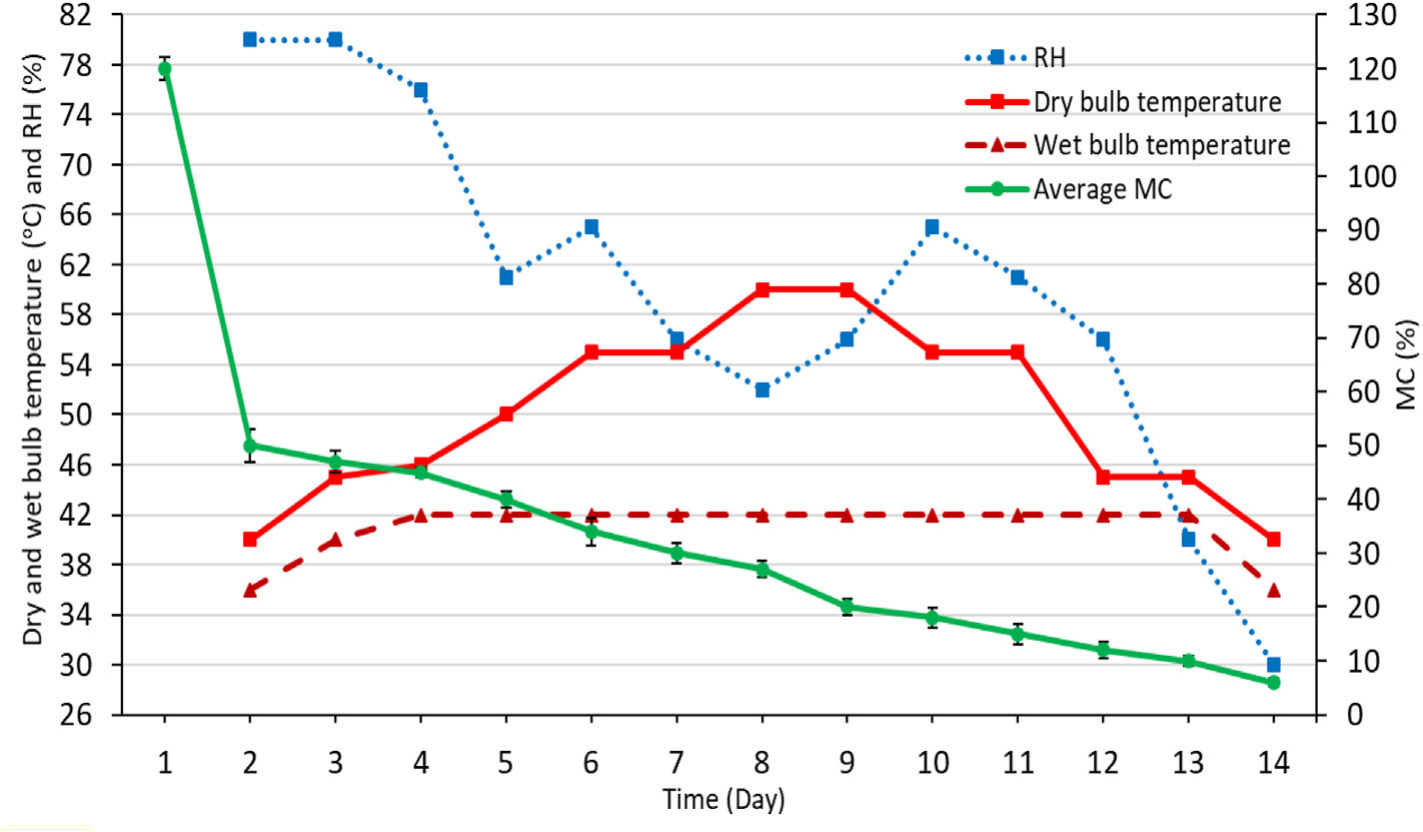
The drying performance of *A. chinensis*. Symbols refer to RH = relative humidity and MC = moisture content.

The MC decreased gradually and the desired MC (6–10%) was achieved from its green condition (120 ± 5%) on day 13 (Figure 1). The MC was decreased with increasing the drying time (DT). The RH, dry bulb temperature, and wet bulb temperature were in the range of 52–80%, 40–60 °C, and 36–42 °C, respectively during the drying. Dry bulb temperature and RH were used in a wider range compared to wet bulb temperature. The wet bulb temperature was more or less stable during the entire drying schedule. The average total DT was 13 days from 120 to 6% of the final average MC. The effect of temperature of dry bulb and wet bulb and RH on DT was not strongly correlated in this study. High dry bulb temperature was applied when the MC of the board was in the range of fiber saturation point to the equilibrium level. However, further studies are needed to consider for a better understanding of their relationship. Generally, the drying characteristics depend on the initial moisture content and density of wood [11]. Drying condition, i.e., high temperature also affects the drying characteristics, the higher the drying temperature the lower the drying time [2, 11]]. A similar kiln drying was carried out on European hophornbeam (*Ostrya carpinifolia* Scop.) lumber in a previous study [11]. The authors used a mild and harsh drying schedule to check the drying characteristics. In the mild drying schedule, the RH, dry bulb temperature, and wet bulb temperature were in the range of 22–89%, 43.3–71.1 °C, and 41.1–61.1 °C, respectively. On the other hand, in a harsh drying schedule, the RH, dry bulb temperature, and wet bulb temperature were in the range of 23–87%, 48.9–75.0 °C, and 45.0–65.0 °C, respectively. The required time to reach 8% MC for mild and harsh drying was about 23 and 21 days, respectively. Frühwald [2] used high temperatures to reduce the drying time of Norway spruce (*Picea abies*) timber during kiln drying. Considering the previous investigations, the drying time of *A. chinensis* was lower. Basic density was 0.33 g/cm^3^. This lower value of basic density might cause the faster drying of *A. chinensis*. Tenorio et al. [23] also have observed the effect of wood density on drying time.

#### 3.1.2 Moisture gradient

The variation of moisture content in the same board (two ends and middle of a single board) for different boards was varied. The variation was ≤1.0% of a single board for the maximum samples (60% of the total samples) while it was >1.0 - ≤5.0% of a single board for 20% of the total samples. The rest of them (remaining 20% samples) showed a variation of >5.0 - ≤9.0% of a single board. It showed better performance in terms of moisture gradient sample percentage. However, the implication of long-term conditioning in the kiln can eliminate the moisture gradient issues of kiln-dried *A. chinensis* board.

### 3.2 Drying quality

#### 3.2.1 Drying defects

Drying defects were observed in the form of a check, twist, and collapse (Table 2). The total proportion of check was observed 22.5% among the total dried boards. A sever check occurred for 16.1% dried lumber. The maximum depth of the check was 4.5 mm. The check was found in the end and surface. For little check was observed on the surface with a maximum depth of 1.0 mm. Twist proportion was 32.5% in lumber. The proportion of collapse occurred for 7.3% of the total dried boards. Drying defects depend on the initial moisture content [5, 23, 24, 25, 26] and pith content [2, 27] of the samples. From this study, it was noticed that 38.1% of the wood pieces consist of knots (dead and alive), and 25.82% consist of pith. Thus, these factors might cause defects in the dried samples. The release of water in vapor causes more pressure for high moisture content wood, and low-density wood is susceptible to internal pressure leading to the collapse. In addition, there was no pre-treatment for the samples and free drying was applied in the current study. In a previous study, Dawson et al. [5] pretreated *Eucalyptus nitens* wood with supercritical CO_2_ before kiln drying to reduce the drying defect. It removed water by lumen water expulsion leading to reducing water tension in subsequent kiln drying. Thus, this technique helped to lower the collapse and washboard depression. In another study, Frühwald [2] applied high-temperature treatment (190 and 210 °C) before kiln drying of Norway spruce wood. The same author used the restraint-drying technique by keeping both ends of samples in a steel frame during drying. The dried timber showed fewer twist defects. The pre-treatment with high temperature reduced the water tension force during kiln drying by removing water and restraint-drying helped to reduce the deformation of wood contributing to the reduction of the defect.

**Table 2:** Drying quality of *A. chinensis* wood samples after drying.

Check (%)		Twist (%)	Collapse (%)	Drying shrinkage rate		
Severe	Little			Maximum shrinkage (%)		
				Tangential	Radial	Volumetric
16.1 (0.70)	6.4 (0.25)	32.5 (1.20)	7.3 (0.15)	15.82 (0.80)	8.42 (0.21)	21.67 (1.05)

Note: Values in parentheses indicate the standard deviation.

#### 3.2.2 Drying shrinkage rate

Dimensional stability was analysed based on the value of shrinkage and it is presented in Table 2. The average volumetric shrinkage was considered as the measurement of the dimensional stability for this study. It was observed 21.67% for *A. chinensis* board. The use of green wood without any types of pre-treatment, i.e., air-drying, might cause the shrinkage in this study. The shrinkage depends on the tree species, drying temperature, and drying methods. The variation of micro- and ultrastructure of the wood might cause the high shrinkage of this species. It can be reduced by controlled drying and pre-treatment [28]. Longer conditioning time can reduce the deformation of the wood [29]. Further studies may help to explore the reason for this finding and the way of shrinkage reduction.

## 4 Conclusions and recommendations

The drying characteristics of *A. chinensis* board was analysed in this study. The results showed that it can be dried to obtain the target moisture content of 6–10% in a shorter time. There were some defects, i.e., check, twist, collapse observed in the dried board. However, the required drying time was found to be 13 days. Further studies are needed to reduce the drying defects keeping the shorter drying time to develop a complete drying schedule for *A. chinensis* wood to apply on an industrial scale.

## Declarations

### Author contribution statement

Devashish Kumar Ghosh: Performed the experiments; Analyzed and interpreted the data; Contributed reagents, materials, analysis tools or data; Wrote the paper.

Md. Azharul Islam: Conceived and designed the experiments; Analyzed and interpreted the data; Contributed reagents, materials, analysis tools or data; Wrote the paper.

Rupak Kumar Ghosh, Santosh Mazumdar: Analyzed and interpreted the data; Wrote the paper.

Atanu Kumar Das: Conceived and designed the experiments; Analyzed and interpreted the data; Wrote the paper.

### Funding statement

This research did not receive any specific grant from funding agencies in the public, commercial, or not-for-profit sectors.

### Data availability statement

Data will be made available on request.

### Declaration of interests statement

The authors declare no conflict of interest.

### Additional information

No additional information is available for this paper.
